# Posterior Segment Intraocular Foreign Body: Extraction Surgical Techniques, Timing, and Indications for Vitrectomy

**DOI:** 10.1155/2016/2034509

**Published:** 2016-11-29

**Authors:** Dante A. Guevara-Villarreal, Patricio J. Rodríguez-Valdés

**Affiliations:** Instituto de Oftalmología y Ciencias Visuales, Escuela de Medicina y Ciencias de la Salud, Tecnológico de Monterrey, Monterrey, NL, Mexico

## Abstract

Ocular penetrating injury with Intraocular Foreign Body (IOFB) is a common form of ocular injury. Several techniques to remove IOFB have been reported by different authors. The aim of this publication is to review different timing and surgical techniques related to the extraction of IOFB.* Material and Methods*. A PubMed search on “Extraction of Intraocular Foreign Body,” “Timing for Surgery Intraocular Foreign Body,” and “Surgical Technique Intraocular Foreign Body” was made.* Results*. Potential advantages of immediate and delayed IOFB removal have been reported with different results. Several techniques to remove IOFB have been reported by different authors with good results.* Conclusion*. The most important factor at the time to perform IOFB extraction is the experience of the surgeon.

## 1. Introduction

Ocular penetrating injury with Intraocular Foreign Body (IOFB) is a common form of ocular injury [1]. It is encountered in 17–41% of open globe injuries. Sixty-six percent of trauma involving IOFB occurs between 21 and 40 years of age. Most common place for injury is work (54–72%), followed by home (30%). Trauma mechanism involves hammering (60–80%), use of power or machine tools (18–25%), and weapon-related injuries (19%) [1–3].

An ocular trauma review from the United States reported visual acuities of worse than 20/200 in 25% of patients who had IOFB injury [1, 2]. Multiple factors can predict poor visual prognosis, including worse initial visual acuity [1, 4, 5], hyphema [1], vitreous hemorrhage [1], uveal prolapse [1], afferent pupillary defect [1, 6, 7], and retinal detachment [1, 8]. The size of the IOFB is another prognostic factor related with the final Best Corrected Visual Acuity (BCVA). Larger IOFB are related with poor final BCVA [2, 4, 9] while smaller IOFB are related with better visual prognosis [4, 6].

Several techniques to remove IOFB have been reported by different authors. The aim of this publication is to review different timing and surgical techniques related to the extraction of IOFB.

## 2. Material and Methods

A PubMed search on “Intraocular Foreign Body,” “Extraction of Intraocular Foreign Body,” “Indications for Extraction of Intraocular Foreign Body,” “Timing for Surgery Intraocular Foreign Body,” “Surgical Technique Intraocular Foreign Body,” and “Prognosis Intraocular Foreign Body” was made. Results were classified according to relevance according to the investigators. Smaller case series were not included. Priority was given to papers describing surgical techniques and their results.

## 3. Results

### 3.1. Timing and Indications for Vitrectomy

Potential advantages of immediate IOFB removal include a possible decrease in risk of endophthalmitis [1], a decrease in the rate of proliferative vitreoretinopathy (PVR), and a single procedure for the patient [1]. Early surgery was not significantly associated with greater visual improvement [10] but had a significant impact on the development of posttraumatic endophthalmitis according to a report by Yeh [1]. On the other hand, delaying IOFB removal may result in improved control of inflammation caused by initial open globe injury, increase of the ability to assess intraocular structures, and the possible development of spontaneous posterior vitreous detachment (PVD) which might make excision of the posterior hyaloid easier [1]. Furthermore, in some cases, corneal edema associated to the site of entry may preclude visualization of the posterior segment and vitrectomy must be delayed until corneal edema disappears or endoscopic surgery may be indicated. Immediate surgical removal should be performed in all eyes with suspected endophthalmitis [4].

During Operation Iraqi Freedom, the time from injury to IOFB removal was 20.6 days (range 0–90) where the source of the IOFB was a propelled explosive (mortar, rocket-propelled grenade, or missile) in 36% patients and a nonpropelled explosive (grenade, mine, car bomb, or improvised explosive device) in 56% patients. There were no cases of endophthalmitis and they concluded that delayed removal may lead to good visual results and may not substantially increase the risk of endophthalmitis in such case of injuries [7]. However high-energy projectiles may heat-up but this heating may not always be enough to sterilize materials, so the probability of developing endophthalmitis is still present [11, 12].

### 3.2. Surgical Techniques

Table 1 shows surgical techniques of extraction of IOFB by different authors, size of IOFB, and interval accident-surgery.

Yuksel et al. performed 23 Gauge Pars Plana Vitrectomy for removal of retained foreign bodies and then enlarged the sclerotomy into a “T” or “L” shaped wound. There is no mention about the size of the enlarged sclerotomy. 36 patients were included in his report. Age was 43.2 ± 10.9 (15–60) years and they were followed up on for 9.4 ± 6.4 (2–27) months. Nine patients were female and 27 were male; right eye was affected in 54.1%. The interval between the injury and surgery was 14 ± 19.4 days (1–120). Seven cases had a primary wound repair in the same procedure. After vitrectomy, the IOFB was removed through the enlarged “T” or “L” shaped sclerotomy with 20 G forceps. Mean preoperative LogMAR BCVA was 1.44 ± 138 (Snellen equivalent 20/550, range 1.00 to 0.00) and mean postoperative LogMAR BCVA at the final visit was 0.78 ± 0.98 (Snellen equivalent 20/120, range 1.00 to 0.00, *p* = 0.007). In ten patients (27.8%) final visual acuity was better than preoperative values. Mean size of IOFB was 5.63 mm. Fibrin reaction was reported in eight (22.2%) patients. Intraocular pressure elevation was detected in 12 (33.3%) patients. All the patients with intraocular pressure elevation had silicone oil as intravitreal tamponade. Four (11.1%) patients with intraocular pressure elevation were controlled with medical therapy and one patient underwent diode laser cyclophotocoagulation. One of eight patients with silicone oil tamponade developed band keratopathy and phthisis bulbi. They concluded that 23-gauge PPV and sclerotomy enlargement for IOFB removal appear to be an effective and safe procedure in management of posterior segment IOFB [13].

Singh et al. reported an alternative approach for selected cases of IOFB in 14 patients with hammer and chisel injury. All the patients were men with mean age 27.62 ± 8.2 range (17–46 years). The entrance wound was located in 4 patients at the limbus, in 7 patients in paracentral cornea, and in 3 patients in central cornea. Only 4 patients had primary repair. All eyes had posttraumatic cataract with capsular rupture. All the foreign bodies were metallic in nature. They underwent 23-gauge vitrectomy; then a self-sealing superior limbal incision was made. 20-gauge diamond coated IOFB forceps were inserted through the limbal wound and foreign body was grasped along its longest dimension and removed through the limbal port. Pars Plana Vitrectomy was completed after removing the posterior hyaloid. Mean preoperative logMAR visual acuity was 1.51 ± 0.93 (Snellen equivalent 20/647) and at 3 months was 0.17 ± 0.18 (Snellen equivalent 20/29). The improvement at 3 months was maintained at 6 and 12 months. Size of IOFB varied from 1 to 5 mm [14]. Postoperative complications included microscopic hyphema and loose blood in vitreous cavity seen in one eye, which resolved with conservative management within the next 7 days. No hypotony, choroidal detachment, or any other complication in the immediate postoperative period was reported. Late complications like IOP rise, epiretinal membranes, and retinal detachment were not seen in this series.

Park et al. described their technique in cases with full-thickness corneal laceration, traumatic cataract with anterior and posterior capsule rupture, and IOFB using a viscoelastic capture of IOFB with DisCoVisc® (Alcon Laboratories, Fort Worth, TX, USA) during 23-gauge MIVS [5]. The technique consists of primary suture of the wound; then they inserted a 23-gauge stiletto blade through the limbus. After this, they inserted a cannula; the anterior chamber was filled with DisCoVisc; phacoemulsification of the lens was performed, and residual cortical material was aspirated. A core vitrectomy and creation of a posterior vitreous detachment were performed; then IOFB was separated completely from all surrounding tissues, and photocoagulation was applied in the retinal tear. The anterior chamber was refilled with DisCoVisc. The IOFB was grasped using intraocular forceps and lifted to the level of the anterior capsule without changing hands. Once in the anterior chamber the IOFB was extracted through the main corneal incision site using the forceps. There were no changing hands, enlarging sclerotomy, or creating a new limbal wound.

Another method for extraction of magnetic IOFB is the external electromagnet that was developed in 1842 by Meyer [15] and their use was to prevent encapsulation [16]. PPV is still having better anatomical and functional prognoses and timely appears to markedly reduce the risk of endophthalmitis development compared with magnet extraction. Extraction of IOFB by electromagnet have 23% risk of developing vitreous hemorrhage by its strong pulling force [17, 18] and 10% risk of developing endophthalmitis [18].

Transscleral removal consisted in extracting an IOFB through sclerotomy without performing a PPV. Rusnak et al. reported 37 eyes with diagnosis of penetrating eye injury where 28 eyes were operated on by PPV and 9 cases by transscleral extraction without PPV. The extraction was performed on days from 1 to 12 (7.2 on average). They performed a conjunctival peritomy then a sclerotomy of 1.5 to 3.0 mm at 4.5 from the limbus. Using an indirect ophthalmoscope and a magnet, the IOFB was attracted. When the IOFB is removed, the sclera was sutured. In all the cases, cryopexy was performed. The initial BCVA was 0.8 (Snellen equivalent 20/25) with a final BCVA of 0.97 (Snellen equivalent 20/20-). There was no report about complications [19].

The Endoscopic Pars Plana Vitrectomy was first described by Thorpe in 1934 where the proposed indication was any media opacity [20]. The advantages of using an endoscopic system are bypassing anterior segment opacities, manipulation, and visualization of anterior structures [20, 21]. Regarding limitations of this technique, it currently does not permit bimanual instrumentation and the postoperative examination is still difficult by anterior segment opacity.

Shah et al. in their model eye study found that the use of perfluorocarbon fluids protects the macula in case of dropping the IOFB while attempting to remove it from the eye, especially when the IOFB has lower mass [22].

### 3.3. Prognostic Factors

Different prognostic factors have been related to a better final visual acuity such as better presenting visual acuity and hammering metal on metal as a mechanism of injury. Poor visual outcome is associated with poor presenting visual acuity [1, 4, 5], presence of afferent pupillary defect [1, 6, 7], vitreous hemorrhage [1], retinal detachment [1, 8], or prolapse of intraocular contents [1]. Greven et al. have not found relationship between size of the IOFB and the visual outcome [2], however, other authors have [4, 6, 9].

The Ocular Trauma Score (OTS) is a useful tool prior to surgical approach for predicting the visual prognosis. It was described by Kuhn et al. [8] in 2002 based on a review of databases of the United States of America and Hungary to identify the predictors of visual acuity after open globe injury (Table 2). To calculate the OTS a numerical value is assigned to the Best Corrected Visual Acuity (BCVA) of the patient. From this score “raw points” are subtracted according to a set of 5 variables. These variables are globe rupture (according to the raw points, this is the worst prognostic factor for final visual acuity even worse than endophthalmitis) [23], endophthalmitis (is not a frequent factor but can be present as soon as 24 hours after the event) [23], perforating injury, retinal detachment, and relative afferent pupillary defect (Table 2). The remaining value determines the Ocular Trauma Score which is stratified into five categories and they reflect the probability of obtaining a range of specific visual acuity at 6 months (Table 3). The use of OTS is an excellent prognostic tool to predict the visual acuity after ocular trauma [8, 23–25].

Agrawal et al. reported a series of 172 eyes with open globe injuries and its correlation to the Ocular Trauma Score. In his series IOFB had no impact on final BCVA. Good initial BCVA was associated with good final vision outcome. When RAPD or vitreous loss was present, more than 50% of patients had final BCVA less than hand motion [24].

## 4. Discussion

Several studies showed that the time to perform a PPV is not a prognostic factor to develop endophthalmitis. Extraction of an IOFB can delayed for long time with adequate vigilance of the patient as was seen in the report during Operation Iraqi Freedom, where the extraction of IOFB was delayed in some cases up to 90 days. We advise to perform immediate extraction of the IOFB in case of suspected endophthalmitis; otherwise it can be delayed for a few days until corneal edema resolves and allows a better visualization during vitrectomy, intraocular inflammation is controlled, and suprachoroidal hemorrhage liquefies and thus can be drained if necessary. These processes usually take between 3 and 14 days.

In this review we compared the results of different IOFB extraction techniques in previous reports. Yuksel et al. reported 36 patients with trauma related to IOFB using the “T” or “L” sclerotomy where the interval between accident and surgery was 14 days. The size of the IOFB was similar in all papers except in the viscoelastic capture but its technique requires a limbal port where IOFB can be extracted regardless of the size. The extraction of IOFB without PPV can be possible as shown by Rusnak et al. where they reported 9 patients with IOFB removed through a transscleral incision using a magnet achieving a good visual acuity and there were no report of complications; however other reports debate about the risk of using this technique. Park reported 10 patients in their paper, but there is no detail about BCVA or interval between accident and surgery since their publication is only a surgical technique description. We do not recommend extracting an IOFB in the vitrectomy era without performing vitrectomy, since there is a risk for vitreous incarceration in the wound and unnecessary traction can be applied to intraocular structures. Furthermore, if there is an intraretinal foreign body, some ocular trauma experts even recommend a retinochoroidectomy around the area of the posterior lesion to reduce postoperative cellular proliferation and consequent traction. The use of perfluorocarbon fluids before lifting the IOFB is highly recommended to try to avoid macular damage in case of dropping. Site and technique of extraction must be decided by the surgeon according to the injuries proper to each case and personal experience. We usually use a limbal approach if the crystalline lens is not present and a scleral enlargement when it is.

The OTS is an effective tool to predict the visual acuity. In the moment of the assessment of a patient with ocular trauma, it is important to perform a detailed examination of the affected area and try to identify any signs related to the OTS because it can give us a prognostic visual acuity in the moment of patient counseling.

## 5. Conclusions

In this review we observed that the IOFB can be removed by any technique regardless of the size of the IOFB. We conclude that PPV should be performed in all patients, and the exact mechanism of removal of IOFB should depend on the surgeon's preference given that all techniques described above were successful. The decision of which technique to use on a particular subject must be decided upon damage of particular eye structures (cornea, lens, and retina).

## Figures and Tables

**Table 1:** Different IOFB extraction techniques.

	Patients	BCVA baseline	BCVA at 3 months	Accident-surgery interval	IOFB size	Extraction technique
Yuksel et al.^*∗*^	36 patients	20/550	20/120	14.2 ± 19.4 days	5.63 mm	“T” or “L” sclerotomy
Singh et al.^*∗*^	14 patients	20/647	20/29	No mention	1 to 5 mm	Translimbal
Park et al.	10 patients	—	—	—	2.75 ± 1.04 mm	Viscoelastic capture
Rusnak et al.^*∗∗*^	9 patients	20/25	20/20-	1 to 12 days	1.5 to 5 mm	Transscleral using magnet

^*∗*^BCVA converted from LogMAR.

^*∗∗*^BCVA converted from decimal.

**Table 2:** The Ocular Trauma Score (OTS). Reproduced from Kuhn et al. [8].

Baseline visual acuity	Raw points	Diagnosis	Raw points	Sum of raw points	OTS
NPL	60	Globe rupture	−23	0–44	1
LP-HM	70	Endophthalmitis	−17	45–65	2
1/200–19/200	80	Perforating injury	−14	66–80	3
20/200–20/50	90	Retinal detachment	−11	81–91	4
>20/40	100	RAPD^*∗*^	−10	92–100	5

^*∗*^Relative afferent pupillary defect (RAPD).

**Table 3:** Conversion of raw points into OTS category and calculating the likelihood of the final visual acuity in five categories. The Ocular Trauma Score (OTS). Reproduced from Kuhn et al. [8].

Sum of raw points	OTS	No Light perception	Light perception/hand motion	1/200–19/200	20/200–20/50	>20/40
0–44	1	74%	15%	7%	3%	1%
45–65	2	27%	26%	18%	15%	15%
66–80	3	2%	11%	15%	31%	41%
81–91	4	1%	2%	3%	22%	73%
92–100	5	0%	1%	1%	5%	94%
